# Anti-Obesity and Gut Microbiota Regulation Effects of Phospholipids from the Eggs of Crab, Portunus Trituberculatus, in High Fat Diet-Fed Mice

**DOI:** 10.3390/md20070411

**Published:** 2022-06-23

**Authors:** Laijin Su, Hongli Zhu, Sichun Chen, Mengyu Du, Xiaofeng Wan, Yishu Liu, Shiwei Hu, Yangli Xu

**Affiliations:** 1 College of Life and Environmental Science, Wenzhou University, Wenzhou 325035, China; sulj@wzu.edu.cn; 2 Zhejiang Provincial Key Laboratory for Water Environment and Marine Biological Resources Protection, Wenzhou University, Wenzhou 325035, China; 3 National Engineering Research Center for Maine Aquaculture, Zhejiang Ocean University, Zhoushan 316022, China; zhuhongli2020@163.com (H.Z.); chensichun2022@163.com (S.C.); dumengyu816@163.com (M.D.); wanxiaofeng1998@163.com (X.W.); liuyishu1016@163.com (Y.L.); 4 Wenzhou Academy of Agricultural Science, Wenzhou Characteristic Food Resources Engineering and Technology Research Center, Wenzhou 325006, China; xuyangli@wzvcst.edu.cn

**Keywords:** phospholipids, composition of fatty acids, *Portunus trituberculatus* eggs, anti-obesity, gut microbiota

## Abstract

There are resourceful phospholipids in the eggs of the crab, *Portunus trituberculatus* (*Pt*-PL). However, their components and bioactivities regarding obesity were unclear. Here, we investigated the composition of *Pt*-PL and their fatty acids. Moreover, its effects on obesity and gut microbiota were also evaluated in high fat diet (HFD)-fed mice. The results showed that *Pt*-PL contained 12 kinds of phospholipids, mainly including phosphatidylcholine (PC, 32.28%), phosphatidylserine (PS, 26.51%), phosphatidic acid (PA, 19.61%), phosphatidylethanolamine (PE, 8.81%), and phosphatidylinositol (PI, 7.96%). Polyunsaturated fatty acids (PUFAs) predominated in the fatty acids components of *Pt*-PL, especially eicosapentaenoic acid (EPA) and docosahexaenoic acid (DHA). Animal experiments demonstrated that *Pt*-PL significantly alleviated body weight gain, adipose gain, hepatic gain, fasting blood glucose, serum insulin, lipid levels in serum and the liver, and systematic inflammation in HFD-fed mice. Furthermore, *Pt*-PL regulated gut microbiota, especially in a dramatic reduction in the ratio of Firmicutes to Bacteroidetes at phylum level, as well as significant amelioration in their subordinate categories. *Pt*-PL reduced fecal lipopolysaccharide and total bile acids, and elevated fecal short chain fatty acid (SCFA) concentrations, particularly acetate and butyrate. These findings suggest that *Pt*-PL possesses anti-obesity effects and can alter gut microbiota owing to the abundance of PUFAs. Therefore, *Pt*-PL may be developed as an effective food supplement for anti-obesity and regulation of human gut health.

## 1. Introduction

Obesity is one of the most serious global health metabolic disorders, with an incidence that increases rapidly every year and is correlated with many chronic diseases, such as hyperlipidemia, hyperglycemia, low-grade inflammation, type 2 diabetes, cardiovascular disease, etc. [1,2]. Therefore, it has been a major challenge to learn how to curb the development of obesity and how to prevent obesity and its related symptoms. Several studies have proved that gut microbiota are major factors increasing incidence of obesity through affecting nutrient digestion, energy metabolism, chronic inflammation, or other metabolic diseases [3,4]. It has been reported that the increased ratio of Firmicutes to Bacteroidetes and disturbance of Akkermansia, Bifidobacterium, Desulfovibrionales, Lactobacillales or other bacteria could alter the development of obesity in humans and rodents [5,6,7]. However, other papers have exhibited the fact that the occurrence of obesity was unrelated to the abundances of Firmicutes and Bacteroidetes [8], suggesting that obesity may be affected by other factors. Moreover, obese mice tend to have a low concentration of short chain fatty acids (SCFAs) and high levels of lipopolysaccharide (LPS) and total bile acids [9,10]. Evidence suggests that treatment with LPS could promote adipocyte differentiation and insulin resistance in mice adipose cells [11], while dietary supplementation with SCFAs could alleviate obesity in HFD mice and regulate gut microbiota [12]. These studies suggested that gut microbiota could administer obesity and its related diseases.

The swimming crab, *Portunus trituberculatus*, is widely distributed in the Western Pacific coast and has been a prominent economic marine product because of its high nutritional value and great production. More than 500,000 tons were produced in China both in 2019 and 2020 [13]. There are several papers that have analyzed the gene sequence or aquaculture of *P. trituberculatus* [14,15], but little research has been conducted on its processing or utilization. Our previous studies showed that egg oil isolated from *P. trituberculatus* contains abundant phospholipids (*Pt*-PL), and the egg oil could improve insulin resistance and mitigate obesity through regulating gut microbiota [16,17]. Recently, we revealed that phosphatidylserine from *P. trituberculatus* eggs (Pt-PS) could improve insulin resistance by activation of the insulin signal pathway and amelioration of gut microbiota dysbiosis [18]. However, it is unclear what the components of the phospholipids in *P. trituberculatus* eggs are and their fatty acid composition. The bioactivity of *Pt*-PL has also not been investigated. Here, we isolated *Pt*-PLs and analyzed their fatty acids composition, and carried out research into their anti-obesity effects and their effects on gut microbiota, with a view to promoting the exploitation and utilization of this marine product.

## 2. Results

### 2.1. The Composition of Phospholipids of Pt-PL and Their Fatty Acids

As shown in Table 1, we detected 12 kinds of phospholipids in *Pt*-PL. The main phospholipids in *Pt*-PL contained phosphatidylcholine (PC, 32.28%), PS (26.51%), phosphatidic acid (PA, 19.61%), phosphatidylethanolamine (PE, 8.81%), and phosphatidylinositol (PI, 7.96%), in which PC accounted for the highest proportion. There were other phospholipids in *Pt*-PL, which all composed less than 1%, except lysophosphatidylcholine 2.87%, such as cardiolipin (CL), phosphatidylglycerol (PG), sphingomyelin (SM), and several lysophospholipids.

We subsequently measured fatty acids composition of the 5 main phospholipids in *Pt*-PL (Table 2). In PA, there were 6 kinds of fatty acids, in which unsaturated fatty acids (UFA) accounted for 95.70% and they were all polyunsaturated fatty acids (PUFA). Uppermost, EPA accounted for 51.84% in the fatty acids of *Pt*-PL, while DHA accounted for 8.95%.

There were 18 kinds of fatty acids in PC, in which UFA accounted for 97.55% and PUFA accounted for 78.66%. Importantly, the fatty acids in PC contained 28.86% EPA and 26.92% DHA.

We detected 13 kinds of fatty acids in PE, and PUFA accounted for 95.65%. The proportion of EPA and DHA was 53.76% and 19.48%, respectively.

In PI, there were only 4 kinds of fatty acids and they were all PUFAs. EPA and DHA accounted for 62.16% in total.

In our previous study, we separated PS from *Pt*-PL. The main composition of fatty acids in PS were UFA (69.29), in which 18.70% was EPA and 30.43% was DHA [18]. Especially notable was the fact that there were high concentrations of bacterial acids (C17:0 and C17:1, 12.50% in total).

### 2.2. Effects of Pt-PL on Obesity

**Figure 1:**
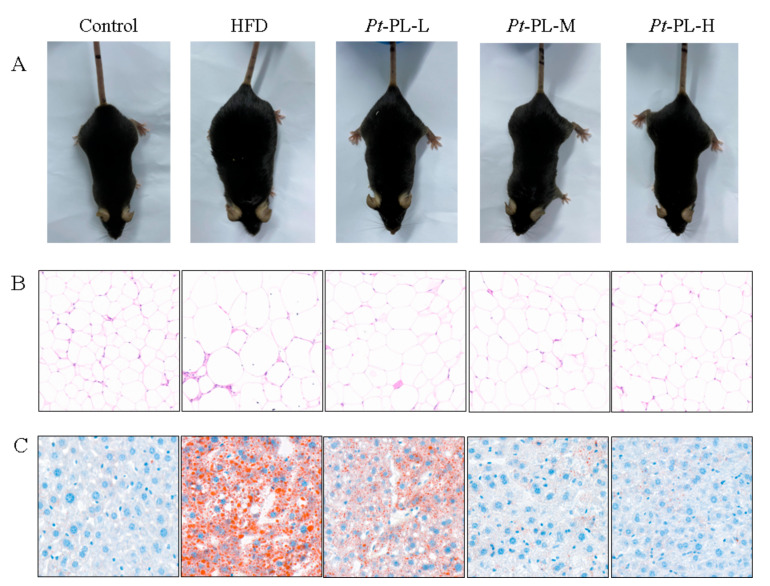
Effects of *Pt*-PL on obesity in HFD-fed mice. (**A**), Mice body type; (**B**), Hematoxylin & eosin strain on the epididymal adipose tissue; (**C**), Oil red O strain on the liver tissue.

In the animal experiment, the food intake of HFD-fed mice was lower than that of control mice, while the calorie intake of HFD animals was higher than that of the control group (*p* < 0.05), which suggested that the HFD-fed mice ingested more calories than the control mice (Table 3). The HFD mice showed an 88.37% increase in body weight gain, compared with that in the control group. When treated with *Pt*-PL, the body weight gain was significantly reduced by 11.97%, 30.58%, and 22.48% in the low, middle, and high dosage groups of the *Pt*-PL groups, compared with the HFD group, respectively (*p* < 0.05). As compared with the control group, the HFD group displayed an evidently higher adipose weight (*p* < 0.05), including perirenal, epididymal, and abdominal subcutaneous adipose. After treatment with *Pt*-PL, the 3 aforementioned fat weights were all significantly decreased in the 3 dosage *Pt*-PL groups (*p* < 0.05). In addition, body weight gain and fat weights in the *Pt*-PL-H group were remarkably lowered compared with those in the *Pt*-PL-L group (*p* < 0.05). Figure 1 A and B show that HFD caused a remarkable increase in body form and epididymal adipose cell size, which were obviously decreased in the three *Pt*-PL groups compared with the HFD group. These results indicated that *Pt*-PL exhibited marked anti-obesity activity.

### 2.3. Effects of Pt-PL on Serum Lipids and Hepatic Lipids

As shown in Table 3, serum TC and TG contents were significantly reduced both in the *Pt*-PL-M and the *Pt*-PL-H groups, compared with the HFD group (*p* < 0.05), while no significant difference occurred between the HFD and *Pt*-PL-L groups. The mice in the 3 *Pt*-PL groups that received dosages all showed significant decreases in serum HDL-C level and increases in serum LDL-C level, compared with HFD-fed mice (*p* < 0.05). Additionally, compared with the *Pt*-PL-L group, serum TC and TG levels were significantly reduced in both the *Pt*-PL-M and *Pt*-PL-H groups (*p* < 0.05), while serum HDL-C concentration obviously increased (*p* < 0.05). However, there were no significant differences in serum LDL-C content among the 3 *Pt*-PL groups. These results indicate that *Pt*-PL can significantly inhibit hyperlipemia in obese mice.

Compared with the HFD group, hepatic weights were significantly reduced by 22.75%, 42.65%, and 46.45% in the 3 *Pt*-PL groups (Table 3, *p* < 0.05), respectively. Hepatic TG content was significantly reduced in the 3 *Pt*-PL groups compared with the HFD group (*p* < 0.05). The mice in the *Pt*-PL-M and *Pt*-PL-H groups displayed marked reduction in hepatic TC levels compared with the HFD group (*p* < 0.05), while there was no significant difference between the *Pt*-PL-L and HFD groups. Furthermore, as shown in Figure 1 C, the lipid droplet number in the liver significantly decreased in the 3 *Pt*-PL groups compared with the HFD group, and the decrease of lipid droplet number in *Pt*-PL-M and *Pt*-PL-H were more obvious than for the *Pt*-PL-L group. These results indicated that *Pt*-PL could decrease lipid accumulation in the liver of obese mice.

### 2.4. Effects of Pt-PL on Blood Glucose, Insulin, and Inflammatory Cytokines

As shown in Table 3, the mice in the 3 *Pt*-PL groups showed significant decreases in fasting blood glucose and serum insulin levels (*p* < 0.05). Interestingly, the reductions of the 2 parameters in the *Pt*-PL-M and *Pt*-PL-H groups were more remarkable than those in the *Pt*-PL-L group (*p* < 0.05).

We also detected inflammatory cytokines in the serum of HFD mice. Table 3 shows that middle and high dosages of *Pt*-PL could significantly lower serum TNF-α concentration (*p* < 0.05), but a low dosage of *Pt*-PL could not. Serum IL-6 level was markedly decreased in the 3 *Pt*-PL groups, compared with HFD mice (*p* < 0.05). Serum IL-1β and IL-10 levels were dramatically reduced and increased in the *Pt*-PL-H group compared with HFD mice, respectively (*p* < 0.05). However, there were no significant changes of the 2 parameters between the *Pt*-PL-L, *Pt*-PL-M, and HFD groups.

### 2.5. Effects of Pt-PL on Gut Microbiota

**Figure 2:**
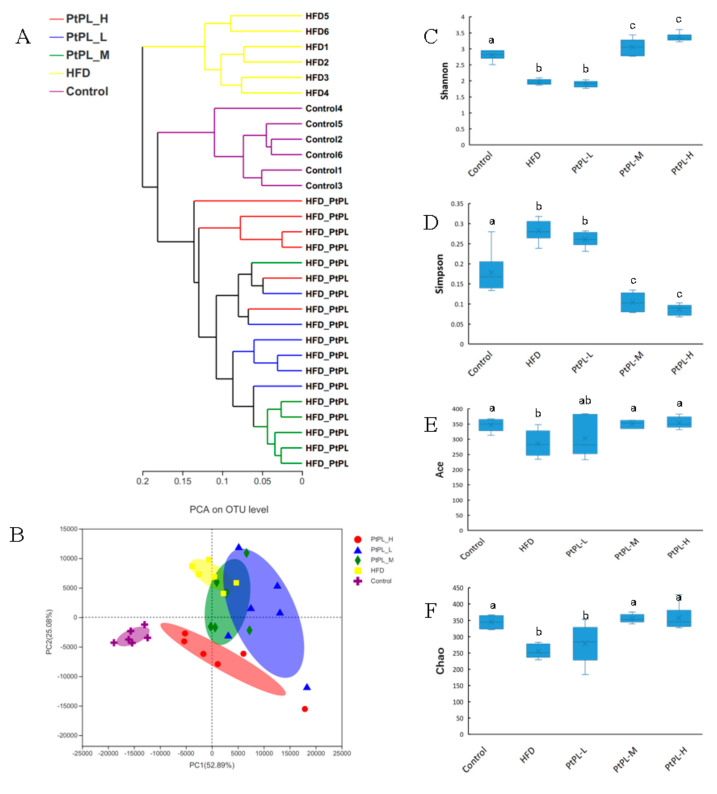
Effects of *Pt*-PL on the composition of the gut microbiota in HFD mice (*n* = 6). (**A**), multivariate analysis of variance from matrix scores; (**B**), the weighted version of UniFrac-based Principal Component Analysis (PCA) on OUT level; (**C**), Shannon index; (**D**), Simpson index; (**E**), Ace index; (**F**), Chao index. Different lowercases represented significant difference (*p* < 0.05) compared between groups.

Obesity is developed and exacerbated once the intestinal bacteria experience dysbiosis in the host. To investigate the effects of *Pt*-PL on maintenance of the microbial community in obese mice, we conducted a V3-V4 of the 16S rRNA gene from the feces. As shown in Figure 2A, the Hierarchical clustering tree on OUT level exhibited distinct separation of the microbial composition between the five groups. The data of PCA methods on the OUT level also showed that the gut microbiota from each group were distinct. Alpha diversity was investigated to express the within-community richness and diversity. Figure 2C,D show significant increase in the Shannon index and decrease in the Simpson index were observed in the *Pt*-PL-M and *Pt*-PL-H groups, compared with the HFD group (*p* < 0.05), implying that the diversity of the gut microbiota obviously increased due to *Pt*-PL treatment. There were significant increases in Ace index and Chao index in *Pt*-PL-M and *Pt*-PL-H groups, compared with HFD group (*p* < 0.05), suggesting that the species richness of the bacteria in the mice was increased by *Pt*-PL treatment. Collectively, the results indicated that the gut microbiota in obese mice was modulated by *Pt*-PL treatment.

**Figure 3:**
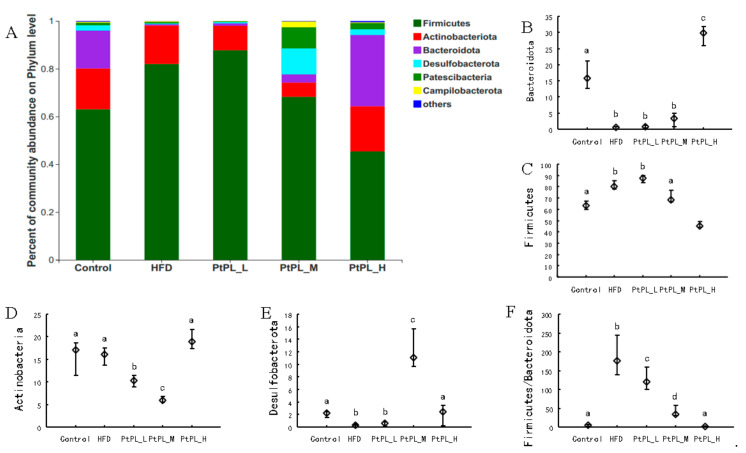
Effects of *Pt*-PL on gut microbiota on Phylum level (*n* = 6). (**A**), Percent of community abundance on Phylum level; (B, Bacteroidetes abundance; (**C**), Firmicutes abundance; (**D**), Actinobacteria abundance; (**E**), Desulfobacterota abundance; (**F**), the ratio of Firmicutes to Bacteroidetes. Different lowercases represent significant difference (*p* < 0.05) between groups.

**Figure 4:**
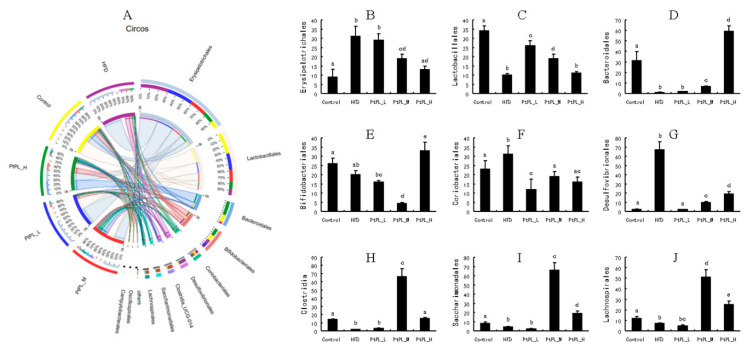
Effects of *Pt*-PL on gut microbiota on order level, using community ternary phase diagram (*n* = 6). (**A**) Percent of community abundance on Order level; (**B**) *Erysipelotrichales* abundance; (**C**) *Lactobacillales* abundance; (**D**) *Bacteroidales* abundance; (**E**) *Bifidobacteriales* abundance; (**F**) *Coriobacteriales* abundance; (**G**) *Desulfovibrionales* abundance; (**H**) *Clostridia* abundance; (**I**) *Saccharimonadales* abundance; (**J**) *Lachnospirales* abundance. Different lowercases represent significant difference (*p* < 0.05) between groups.

The microbial composition in the 5 groups was analyzed at various taxonomic levels. Figure 3 shows the changes of gut microbiota at the phylum level. HFD induced an obvious increase in Firmicutes abundance (*p* < 0.05), and a decrease in the abundances of Bacteroidota and Desulfobacterota (*p* < 0.05). A high dosage of *Pt*-PL could significantly recover these detrimental changes (*p* < 0.05). The middle dosage of *Pt*-PL also caused a significant reduction in Firmicutes abundance and an obvious increase in Desulfobacterota abundance (*p* < 0.05), but no marked changes in Bacteroidota, compared with HFD-fed mice. There were no remarkable differences in the 3 bacteria between *Pt*-PL-L and HFD groups. Interestingly, the mice in the 3 *Pt*-PL groups displayed significant decreases in the ratio of Firmicutues to Bacteroidota (*p* < 0.05). In addition, there was obvious reduction in Actinobacteriota abundance in *Pt*-PL-L and *Pt*-PL-M groups, compared with HFD group (*p* < 0.05), but no significant changes between *Pt*-PL-H and HFD groups. Actinobacterota is a large bacterial community. It contains both beneficial bacteria and harmful ones, such as *Bifidobacterium* and others. In the *Pt*-PL-L and *Pt*-PL-M groups, the Actinobcteriota abundance decreased, which may be associated with reductions of *Bifidobacterium* and other bacteria (Figure 4).

Figure 4 shows the community ternary phase diagram and the relative microbial abundances at the order level. Compared with HFD-fed mice, the animals treated with middle and high dosages of *Pt*-PL showed significant decreases in *Erysipelotrichales*, *Coriobacterials*, and *Desulfovibrionales*, but increases in *Bacteroidales*, *Clostridia*, *Saccharimonadales*, and *Lachnospirales* (*p* < 0.05). The abundance of *Lactobacillales* between HFD and *Pt*-PL-H groups did not show change. However, there was significant increase in the *Pt*-PL-L and *Pt*-PL-M groups, compared with the HFD group (*p* < 0.05). *Bifdobacteriales* abundance was significantly increased in the *Pt*-PL-H group, while it decreased in the *Pt*-PL-M group, compared with the HFD group (*p* < 0.05). Moreover, a low dosage of *Pt*-PL also obviously reduced the abundances of *Coriobacteriales* and *Clostridia* (*p* < 0.05).

**Figure 5:**
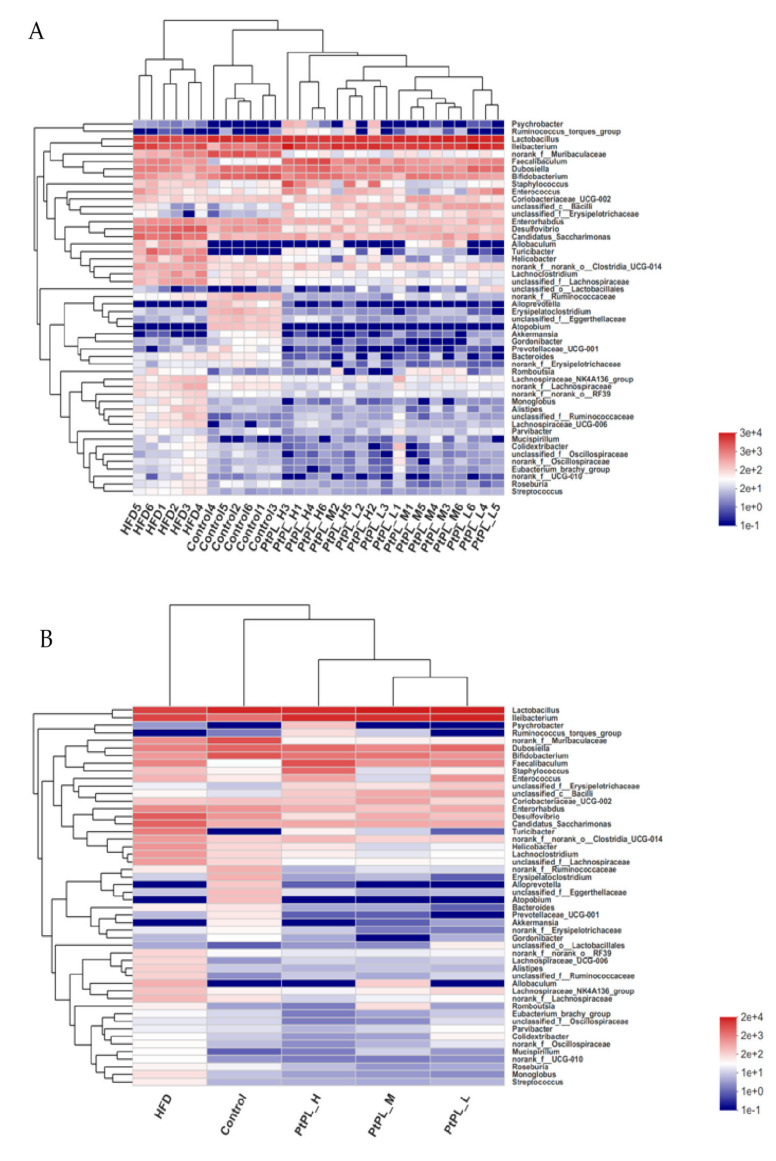
Effects of *Pt*-PL on gut microbiota on the genus level (*n* = 6). Heatmap indicates relative contribution of the top 50 dominant genera in each sample (**A**) and different groups (**B**). The heatmap is color-coded based on row Z-scores.

Next, the relative abundances of bacteria genus were tabulated on a heat map to determine the relative microbial abundances of the gut microbiota (Figure 5). All 50 genera experienced significant difference between the control group and HFD group, while 45 genera different between the HFD group and *Pt*-PL-H, suggesting that the protective effects of *Pt*-PL on mice obesity may be mediated by bacterial subsets of different genera. The number of *norank_f_Ruminococcaceae*, *Erysipelatoclostridium*, and *unclassified_f_Ruminococcaceae*, belonging to Firmicutes, was lower in *Pt*-PL-H-treated mice compared with the HFD group. Moreover, bacteria from other genera, which are positively correlated with obesity, also decreased in the *Pt*-PL-H-treated obese mice, such as *Coriobacteriaceae_UCG-002*, *Desulfovibro*, *Romboutsia*, *norank_f_norank_o_Clostridia_UCG-014*, *unclassified_f_Lachnospiraceae*, *norank_f_Oscillospiraceae*, *unclassified_f_Oscillospiraceae*, *Lachnospiraceae_UCG-006*, *Lachnoclostridium*, *Lachnospiraceae_NK4A136_group*, and *norank_f_Lachnospiraceae*. SCFA-producing microbiota *Lactobacillus*, *Dubosiella*, *Akkermansia*, *unclassified_c_Bacilli*, and *unclassified_o_Lactobacillales* increased in HFD-mice supplemented with a high dosage of *Pt*-PL.

All these results provided strong evidence that *Pt*-PL could regulate gut microbiota in obese mice.

### 2.6. Effects of Pt-PL on LPS, Total Bile Acids, and SCFAs Concentrations

As shown in Table 4, HFD caused significant increase in fecal LPS and total bile acid levels compared with the control mice (*p* < 0.05), while supplementation of different dosages of *Pt*-PL lowered HFD-induced LPS and total bile acid appearance in the feces (*p* < 0.05). Interestingly, the *Pt*-PL-H group showed significant reduction in LPS and total bile acid levels compared with *Pt*-PL-L and *Pt*-PL-M groups (*p* < 0.05). Moreover, fecal acetate, propionate, butyrate, isobutyrate, valerate, isovalerate, and hexanoate concentrations were all significantly increased in both the *Pt*-PL-M and *Pt*-PL-H groups, compared with the HFD group (*p* < 0.05). Butyrate and isobutyrate concentrations were also increased in *Pt*-PL-L group, compared with the HFD group (*p* < 0.05), while other SCFAs had no significant changes. These results indicated that *Pt*-PL regulated secondary metabolites of gut microbiota in obese mice.

## 3. Discussion

Marine phospholipids have increasingly attracted attention on account of their abundant omega-3 fatty acid contents and high biological activities [19]. Previous studies have demonstrated that marine phospholipids could reduce body weight gain and improve hyperglycemia and hyperlipidemia in obese animals [20,21]. Our study analyzed the composition of phospholipids and their fatty acids, and also proved that *Pt*-PL prevented dietary-induced obesity and its associated disorders by modulating the gut microbiota.

It is reported that shrimp head phospholipids contain 53.62% of PC, 30.25% of LPC, 4.48% of PI, 5.06% of PE, and 0.73% of SM [22]. There were 85.24% of PC, 9.32% of PE, 4.75 of SM, 0.16% of PS, and 0.03% of PI in the phospholipids from the head of the shrimp, *Penaeus vannamei* [23]. Liang et al. reported that PC, LPC, PE, and PI accounted for 76.36%, 12.30%, 9.12%, and 1.09% in the total phospholipids from fish roe, *Pseudosciaena crocea*, respectively [24]. PC was the highest component in the phospholipids from marine species, which was alike our data (32.28% of PC in *Pt*-PL). Interestingly, our results showed that there were abundant PS and PA in *Pt*-PL (36.51% and 19.61%, respectively), while the two components were low in other marine phospholipids. On the other hand, the fatty acids compositions of *Pt*-PL were also advantageous, compared with those of several marine phospholipids. For example, the percentage of PUFA accounted for 43.00% of the phospholipids from *Pseudosciaena crocea* roe, among which considerable amounts of DHA and EPA were found 41.90% in total [24]. Saliu et al. reported that PUFA amount was 28.8–33.0% in the phospholipids from the roe of the fish, *Silurus glanis*, with DHA (14.4–15.9%) and EPA (5.8–7.0%) [25]. Ahmmed et al. reported that the phospholipids from the fish roe, *Scomber australasicus*, had 47.0% of PUFA, with 11.3% of EPA and 27.5% of DHA [26]. Our data exhibited that *Pt*-PL contained 75.78% of PUFA with 54.62% of DHA and EPA in total. These results suggested that *Pt*-PL was distinct and advantageous compared with the phospholipids from other marine animals.

Dietary supplementation of *Pt*-PL significantly decreased insulin resistance, lipid levels in serum and the liver, fat accumulation, and systematic inflammation, which are considered the four main pathological phenotypes of obesity, in HFD-fed mice [27]. This confirmed the beneficial activities of *Pt*-PL on obesity. However, energy uptake was not significantly changed between HFD and *Pt*-PL-treated mice, which implied that the anti-obesity activities of *Pt*-PL were not performed through altering energy efficiency. Some papers strongly support our conclusion [28]. Moreover, several studies have also proved that marine phospholipids exert anti-obesity effects. For example, Lu et al. reported that phospholipids from large yellow croaker roe could regulate lipid metabolism, and improve gut microbiota disorder in HFD-fed rats [29]. Phospholipids from krill oil were proved to lower body weight gain, mesenteric adipose tissue, and hepatic lipid content [30].

In light of the fact that obesity is associated with gut microbiota dysbiosis that disturbs the energy homeostasis and glucose metabolism of the host [31], gut microbiota has been considered an important environmental factor in the pathogenesis of obesity and its related diseases [32]. Microbial diversity and species richness are considered new biomarkers of health [33]. *Pt*-PL could manipulate the gut microbiota toward a healthy composition, since our results indicated remarkably improved gut bacterial diversity and richness in HFD-fed mice, was demonstrated by the correlation analysis in this study. The increased ratio of Firmicutes to Bacteroidetes, and modulating the abundances of these major phyla, has been recognized as an important indicator to potential prevention of obesity [34]. A similar result was found in our study, implying that *Pt*-PL may produce anti-obesity activities in HFD mice by reversing the ratio of Firmicutes to Bacteroidetes. Moreover, the abundance of the genus *norank_f_Ruminococcaceae*, *Erysipelatoclostridium*, and *unclassified_f_Ruminococcaceae*, which was highly positively correlated with obesity and obesity-related disease [35], was down-regulated by *Pt*-PL. Nevertheless, intriguingly, the levels of genus bacteria, which were previously reported to be negatively linked to weight reduction or a lean phenotype, were enriched by *Pt*-PL, such as and *Akkermansia* [7]. These findings indicate that *Pt*-PL can produce anti-obesity effects by modifying some specific bacterial phylotypes.

Apart from the above-mentioned specific gut microbiota, the SCFAs-producing bacteria phylotypes were also enriched by *Pt*-PL supplementations in HFD-fed mice, including *Lactobacillus*, *Dubosiella*, *unclassified_c_Bacilli*, and *unclassified_o_Lactobacillales* [36]. Significantly, the elevations of SCFAs-producing gut microbiota were positively correlated with increased concentrations of SCFAs in the feces of the mice treated with *Pt*-PL, particularly acetate and butyrate. SCFAs are hugely helpful to alleviate glucose tolerance and insulin resistance by beneficially modulating liver and adipose tissue function [37]. Furthermore, our study also proved that fecal LPS and total bile acid levels, which can lead to inflammation-dependent adiposity and insulin resistance in obese mice [38], were significantly reduced by *Pt*-PL treatment in HFD-fed mice. These changes were associated with a decrease in LPS-producing microbiota, such as order *Desulfovibrionales* and genus Desulfovibrio [39], and improvement in fat weight, lipid levels, inflammatory cytokine levels, and insulin resistance. Thus, the better anti-obesity effects of *Pt*-PL could be explained by the enrichment of beneficial bacteria with their metabolites and the attenuation of destructive bacteria abundance with their metabolites.

## 4. Materials and Methods

### 4.1. Preparation of Pt-PL and Their Fatty Acids Composition

Total lipids were extracted from *Pt* eggs according to our previous paper [18], and subsequently used to isolate phospholipids. Briefly, fresh *Portunus trituberculatus* eggs, early-stage embryos, were freeze dried, and subsequently homogenized in chloroform-methanol to gain total lipids.

Phospholipids were subsequently isolated using the lipids by column chromatography on silica gel. The composition of phospholipids was detected using high performance liquid chromatography (HPLC, 1200, Agilent, Santa Clara, CA, USA) with an ELSD evaporative light-scattering detector (ELSD2000, Alltech, Chicago, IL, USA). Briefly, gradient elution was used to analyze the phospholipids as: chloroform:methanol:ammonium hydroxide (eluant A, 80:19.5:0.5, *v/v/v*) for 6 min, chloroform:methanol:water:ammonium hydroxide (eluant B, 60:34:0.5:5.5, *v/v/v/v*) for 10 min, and eluant A for 20 min with a flow velocity 1.0 mL/min. ELSD conditions were: nitrogen, 4.5 MPa, flow rate 2.0 L/min, drift tube temperature 65 °C.

The phospholipids were esterified with 2 mol/L KOH in methanol at 80 °C for 1 h. After the esterification reaction, the upper organic layer was diluted with n-hexane after centrifugation at 4500× *g* for 15 min, and traces of water were subsequently removed with Na_2_SO_4_, filtered using a 0.22 µm filter, and preserved at −20 °C under the protection of nitrogen, before analysis for fatty acid composition [40]. The fatty acid composition was analyzed using gas chromatography (GC, 7820A, Agilent, Santa Clara, CA, USA) equipped with a hydrogen flame ionization detector (FID) and a FFAP-fused silica capillary column (30 m × 0.53 mm, 1 μm). GC conditions were: oven temperature, 50 to 100 °C (10 °C/min, 1 min), 100 to 150 °C (5 °C/min, 5 min), and 150 to 200 °C (20 °C/min); nitrogen (carrier gas), 1 mL/min; FID and injector temperature, 250 °C; injection volume, 2.0 μL. Fatty acids were identified by comparing the retention times of the sample peaks with those of a mixture of fatty acid methyl ester standards. The contents of the fatty acids were expressed as the weight percentage (% *w*/*w*) of the total fatty acids detected with chain lengths of 4–23 carbon atoms.

### 4.2. Animal Experiments

Male C57BL/6J mice (licensed ID: SCXK2019-0001), 16–18 g, were purchased from Vital River Laboratory Animal Center (Beijing, China). The mice were housed in individual cages under a 12-h light/dark cycle environment daily. The animals (50 mice in total and 10 per group) were stratified and randomized into 5 groups: control group (normal chow diet: 70% carbohydrate, 20% protein, and 10% fat), HFD group (HFD: 29% carbohydrates, 16% protein, and 55% fat), low dosage of *Pt*-PL group (*Pt*-PL-L, HFD + 1% *Pt*-PL), medium dosage of *Pt*-PL group (*Pt*-PL-M, HFD + 1.5% *Pt*-PL), and high dosage of *Pt*-PL group (*Pt*-PL-L, HFD + 2% *Pt*-PL). Body weight and food intake were recorded every week. After 18 weeks of feeding, each animal was housed in separated metabolic cages for 24 h to collect feces. Subsequently, the mice were sacrificed after fasting 5 h. Blood was collected to test glucose, lipid, and inflammatory parameters. The liver and adipose tissues were stripped for further study.

### 4.3. Blood Glucose, Lipids, Insulin, and Inflammatory Cytokines Test

At the end of the animal experiments, 5-h fasted mice were sacrificed to collect blood. Fasting blood glucose and serum lipids (including TG, TC, HDL-C, and LDL-C) levels were determined using commercial testing kits (Biosino, Beijing, China). Serum insulin and inflammatory cytokines (including TNF-α, IL-6, IL-1β, and IL-10) levels were determined using insulin ELISA kits (Invitrogen, Carlsbad, CA, USA).

### 4.4. Hepatic TG and TC Contents Detection

The liver tissues (0.25 g) were homogenized using chloroform-methanol (2:1, *v/v*) to gain total lipids. The lipids were suffered with water to remove water-soluble fraction, subsequently with light petroleum to remove water, and finally freeze-dried. The dried lipids were dissolved in isopropanol-polysorbate100 (9:1, *v/m*) and then measured TG and TC contents using commercial testing kits (Biosino, Beijing, China).

### 4.5. Hematoxylin and Eosin (H&E) and Oil Red O Staining

The epididymal adipose and liver tissues were peeled rapidly from the animals, subsequently fixed in 10% formalin, paraffin embedded, sectioned, and finally stained with H&E (epididymal adipose tissue) or oil red O (liver tissue). The epididymal adipose and liver microscopic structures were observed and photographed using a fluorescence microscope (Eclipse Ci, Nikon, Japan).

### 4.6. Gut Microbiota Analysis

Fecal DNA (*n* = 6 per group) was extracted using DNA gel extraction kit (Axygen, Silicon valley, CA, USA). DNA amplify, sequences analysis, taxonomic identification, alpha and beta diversities, and LEfSe analysis were performed according to our pervious stidies [18].

### 4.7. Fecal LPS, Total Bile Acids, and SCFAs Determination

Faeces were homogenized in ice-cold Millipore H2O and then centrifuged. The supernatant was heated to inactivated proteins. Fecal LPS and total bile acids were measured using ELISA kits (Invitrogen, Carlsbad, CA, USA).

Fecal SCFAs levels were evaluated using GC-MS (QP2010-Ultra, Shimadzu, Tokyo, Japan) according to our pervious study [18].

### 4.8. Statistical Analysis

Data were expressed as the means ± standard deviation and evaluated using a one-way analysis of variance followed by Duncan’s test using SPSS version 17.0 (SPSS Inc., Chicago, IL, USA). Statistically significance was set at *p* < 0.05.

For gut bacterial data, Shannon, Simpson, Ace, and Chao indexes were verified using Student’s *t*-test. Univariate differential abundance of OUT at phylum, order, and Genus levels was analyzed by incorporating Fisher’s test. Statistically significance was considered *p* < 0.05.

## 5. Conclusions

The results show *Pt*-PL contains 13 kinds of phospholipids, including PC, PS, PA, PE, and PI. PUFAs predominate in the fatty acid components of *Pt*-PL, especially EPA and DHA. *Pt*-PL significantly alleviated HFD-induced obesity and obesity-related disorders in mice, such as insulin resistance, lipid accumulation, and inflammation. These activities were directly related with the modulation of the gut microbiota community and their metabolites. Our findings offer new insights into the anti-obesity effect of dietary *Pt*-PL as an alternative novel marine functional ingredient.

## Figures and Tables

**Table 1:** The composition of phospholipids in *Pt*-PL.

Phospholipids	Proportion (%)	Phospholipids	Proportion (%)
LPC	2.87 ± 0.15	LPE	0.27 ± 0.03
LPA	0.19 ± 0.03	LPS	0.07 ± 0.01
PA	19.61 ± 2.34	PC	32.28 ± 5.87
PE	8.81 ± 1.04	PG	0.22 ± 0.04
PI	7.96 ± 0.83	PS	26.51 ± 4.59
SM	0.87 ± 0.06	Others	0.49 ± 0.07
CL	0.07 ± 0.02		

Data are presented as means ± SD (*n* = 6). CL, cardiolipin; LPC, lysophosphatidylcholine; LPE, lysophosphatidylethanolamine; LPA, lysophosphatidylinositol; LPS, lysophosphatidylserine; PA, phosphatidic acid; PC, phosphatidylcholine; PE, phosphatidylethanolamine; PG, phosphatidylglycerol; PI, phosphatidylinositol; PS, phosphatidylserine; SM, sphingomyelin.

**Table 2:** The composition of fatty acids of main phospholipids in *Pt*-PL.

*Fatty acids*	Proportion (%)				
	PA	PC	PE	PI	PS [18]
C15:0	---	---	---	---	0.59 ± 0.08
C16:0	---	0.62 ± 0.26	---	---	10.95 ± 1.57
C16:1 n-9	---	0.91 ± 0.68	---		---
C16:1 n-7	---	---	0.10 ± 0.06		0.83 ± 0.01
C17:0	---	---	0.03 ± 0.01	---	5.18 ± 0.84
C17:1 n-7	---	---	---	---	7.32 ± 0.86
C17:3 n-9	---	---	---	---	0.12 ± 0.01
C18:0	4.30 ± 1.03	---	---	---	3.45 ± 0.61
C18:1 n-9	---	0.22 ± 0.03	1.77 ± 0.65	---	1.16 ± 0.22
C18:1 n-7	---	0.80 ± 0.16	1.05 ± 0.34	---	0.71 ± 0.14
C18:2 n-6	---	0.50 ± 0.01	0.11 ± 0.05	0.20 ± 0.07	0.59 ± 0.01
C18:3 n-3	---	---	---	---	0.50 ± 0.02
C20:0	---	0.70 ± 0.13	0.12 ± 0.03	---	2.01 ± 0.54
C20:1 n-9	---	1.43 ± 0.39	1.07 ± 0.36	---	1.56 ± 0.39
C20:2 n-6	---	2.26 ± 0.19	0.34 ± 0.11	---	---
C20:3 n-6	3.38 ± 1.01	1.01 ± 0.23	1.54 ± 0.99	---	2.49 ± 0.27
C20:4 n-6	20.11 ± 6.07	13.14 ± 2.80	8.27 ± 1.37	16.82 ± 3.36	0.44 ± 0.09
C20:4 n-3	3.52 ± 0.56	---	---	20.83 ± 4.40	1.16 ± 0.25
C20:5 n-3	51.84 ± 6.04	28.86 ± 6.01	53.76 ± 10.83	24.46 ± 5.31	18.70 ± 3.32
C22:0	---	0.58 ± 0.13	---	---	---
C22:1 n-9	---	15.53 ± 3.67	---	---	---
C22:2 n-6	7.90 ± 4.71	2.41 ± 0.46			2.70 ± 0.56
C22:3 n-6	---	0.60 ± 0.16	---	---	---
C22:4 n-6	---	2.14 ± 0.57	3.96 ± 1.13	---	---
C22:5 n-6	---	0.82 ± 0.19	6.54 ± 0.95	---	---
C22:5 n-3	---	---	1.66 ± 0.33	---	---
C22:6 n-3	8.95 ± 5.33	26.92 ± 5.60	19.48 ± 3.25	37.69 ± 4.79	30.43 ± 2.08
C23:1 n-6	---	---	---	---	0.60 ± 0.11
C23:3 n-6	---	0.02 ± 0.00	---	---	---
Others	0.00 ± 0.00	0.55 ± 0.00	0.21 ± 0.06	0.00 ± 0.00	8.52 ± 1.40
∑SFA	4.30 ± 1.03	1.90 ± 0.17	0.15 ± 0.02	0.00 ± 0.00	22.19 ± 3.15
∑UFA	95.70 ± 34.76	97.55 ± 0.84	99.64 ± 1.59	100 ± 8.96	69.29 ± 5.20
∑MUFA	0.00 ± 0.00	18.89 ± 1.23	3.99 ± 0.44	0.00 ± 0.00	12.16 ± 2.24
∑PUFA	95.70 ± 34.76	78.66 ± 1.07	95.65 ± 1.94	100 ± 8.96	57.13 ± 5.63
∑(EPA+DHA)	60.79 ± 18.89	55.78 ± 5.81	73.24 ± 7.45	62.16 ± 5.35	50.13 ± 2.81

Data are presented as means ± SD (*n* = 6). Fatty acids were analyzed by gas chromatography. MUFA, monounsaturated fatty acids; PA, phosphatidic acid; PC, phosphatidylcholine; PE, phosphatidylethanolamine; PI, phosphatidylinositol; PS, phosphatidylserine; PUFA, polyunsaturated fatty acids; SFA, saturated fatty acids; UFA, unsaturated fatty acids.

**Table 3:** Effect of *Pt*-PL on body weight, blood glucose, insulin, serum lipids, hepatic lipids, and serum inflammatory cytokines in HFD-fed mice.

	Control	HFD	*Pt*-PL-L	*Pt*-PL-M	*Pt*-PL-H
Food intake (g/w)	29.74 ± 2.37 ^a^	25.43 ± 2.19 ^b^	25.62 ± 2.30 ^b^	25.18 ± 3.21 ^b^	25.35 ± 2.82 ^b^
Calorie intake (kcal/w)	121.79 ± 10.08 ^a^	166.82 ± 11.55 ^b^	168.07 ± 12.43 ^b^	165.18 ± 15.26 ^b^	166.29 ± 14.04 ^b^
Body weight gain (g)	11.44 ± 0.48 ^a^	21.55 ± 1.18 ^b^	18.97 ± 0.64 ^c^	14.96 ± 1.09 ^d^	14.55 ± 0.18 ^d^
Perirenal fat weight (g)	0.17 ± 0.03 ^a^	0.95 ± 0.08 ^b^	0.49 ± 0.07 ^c^	0.40 ± 0.08 ^cd^	0.25 ± 0.04 ^ad^
Epididymal fat weight (g)	0.49 ± 0.10 ^a^	4.09 ± 0.54 ^b^	1.84 ± 0.50 ^c^	1.17 ± 0.62 ^cd^	0.99 ± 0.11 ^d^
Abdominal subcutaneous fat weight (g)	0.23 ± 0.05 ^a^	1.17 ± 0.38 ^b^	0.73 ± 0.17 ^c^	0.56 ± 0.11 ^cd^	0.44 ± 0.10 ^d^
Hepatic weight (g)	1.01 ± 0.07 ^a^	2.11 ± 0.29 ^b^	1.63 ± 0.15 ^c^	1.21 ± 0.10 ^cd^	1.13 ± 0.12 ^ad^
Fasting blood glucose (mmol/L)	7.84 ± 0.38 ^a^	15.19 ± 0.67 ^b^	11.08 ± 0.50 ^c^	8.56 ± 0.46 ^d^	7.74 ± 0.37 ^d^
Serum insulin (mU/L)	26.00 ± 0.99 ^a^	32.64 ± 1.09 ^b^	28.79 ± 0.30 ^c^	26.60 ± 1.38 ^ad^	25.95 ± 2.05 ^ad^
Serum TG (mmol/L)	1.38 ± 0.05 ^a^	2.66 ± 0.12 ^b^	2.30 ± 0.13 ^b^	2.09 ± 0.06 ^c^	1.46 ± 0.09 ^a^
Serum TC (mmol/L)	3.79 ± 0.07 ^a^	7.25 ± 0.31 ^b^	5.66 ± 0.47 ^b^	4.54 ± 0.28 ^c^	4.06 ± 0.24 ^a^^c^
Serum HDL-C (mmol/L)	1.19 ± 0.20 ^a^	0.32 ± 0.04 ^b^	0.52 ± 0.05 ^c^	0.71 ± 0.11 ^d^	1.15 ± 0.06 ^a^
Serum LDL-C (mmol/L)	2.50 ± 0.20 ^a^	5.26 ± 0.37 ^b^	3.56 ± 0.30 ^c^	3.28 ± 0.29 ^c^	3.01 ± 0.14 ^ac^
Hepatic TG (mg/g)	20.56 ± 2.14 ^a^	36.28 ± 2.87 ^b^	30.01 ± 2.35 ^c^	28.28 ± 1.98 ^cd^	25.37 ± 2.17 ^d^
Hepatic TC (mg/g)	3.55 ± 0.28 ^a^	5.62 ± 0.41 ^b^	5.05 ± 0.39 ^bc^	4.46 ± 0.40 ^c^	4.38 ± 0.36 ^c^
Serum TNF-α (pg/mL)	56.08 ± 2.87 ^a^	106.01 ± 13.82 ^b^	94.73 ± 6.03 ^b^	76.99 ± 6.43 ^c^	56.94 ± 2.46 ^d^
Serum IL-6 (pg/mL)	57.60 ± 5.45 ^a^	83.46 ± 6.32 ^b^	66.77 ± 4.17 ^c^	60.04 ± 5.86 ^ac^	56.49 ± 4.31 ^a^
Serum IL-1β (pg/mL)	49.28 ± 3.00 ^a^	81.20 ± 4.27 ^b^	80.78 ± 4.16 ^b^	75.31 ± 5.36 ^b^	65.52 ± 2.79 ^c^
Serum IL-10 (pg/mL)	271.86 ± 8.90 ^a^	240.43 ± 7.74^b^	248.74 ± 7.28 ^b^	254.15 ± 10.12 ^b^	272.16 ± 8.51 ^a^

Data are presented as mean ± S.D. (*n* = 10). Multiple comparisons were done using one way ANOVA. Different lowercases represented significant difference (*p* < 0.05) compared between groups.

**Table 4:** Effect of *Pt*-PL on fecal LPS, total bile acids, and SCFAs in HFD-fed mice.

Parameters		Control	HFD	*Pt*-PL-L	*Pt*-PL-M	*Pt*-PL-H
LPS (EU/g faeces)		6.40 ± 0.67 ^a^	13.24 ± 0.98 ^b^	11.58 ± 1.04 ^c^	11.03 ± 0.75 ^c^	6.72 ± 0.54 ^a^
Total bile acids (µmol/g faeces)		6.57 ± 0.62 ^a^	13.34 ± 0.85 ^b^	9.14 ± 0.76 ^c^	8.24 ± 0.59 ^c^	6.89 ± 0.71 ^a^
SCFAs (mg/g faeces)	Acetate	17.63 ± 1.44 ^a^	6.09 ± 0.89 ^b^	6.08 ± 0.77 ^b^	14.51 ± 1.14 ^c^	14.93 ± 1.34 ^c^
	Propionate	5.03 ± 0.34 ^a^	0.62 ± 0.05 ^b^	0.67 ± 0.05 ^b^	1.40 ± 0.09	2.35 ± 0.21 ^d^
	Butyrate	3.69 ± 0.31 ^a^	0.21 ± 0.01 ^b^	0.42 ± 0.02 ^c^	1.80 ± 0.12 ^c^	8.30 ± 0.87 ^d^
	Isobutyrate	0.33 ± 0.02 ^a^	0.02 ± 0.00 ^b^	0.08 ± 0.01 ^c^	0.24 ± 0.02 ^c^	0.35 ± 0.03 ^a^
	Valerate	0.61 ± 0.04 ^a^	0.04 ± 0.01 ^b^	0.04 ± 0.00 ^b^	0.08 ± 0.00 ^c^	0.25 ± 0.01 ^d^
	Isovalerate	0.42 ± 0.03 ^a^	0.12 ± 0.00 ^b^	0.11 ± 0.01 ^b^	0.29 ± 0.02 ^c^	0.28 ± 0.01 ^c^
	Hexanoate	0.10 ± 0.00 ^a^	ND	ND	0.01 ± 0.00 ^b^	0.02 ± 0.00 ^c^
	Total SCFAs	27.76 ± 2.04 ^a^	7.09 ± 0.63 ^b^	7.41 ± 0.65 ^b^	18.33 ± 1.70 ^c^	26.49 ± 2.18 ^a^

Data are presented as mean ± S.D. (*n* = 6). Multiple comparisons were done using one way ANOVA. LPS, lipopolysaccharides; ND, no detection; SCFAs, short chain fat acids. Different lowercases represent significant difference (*p* < 0.05) between groups.
